# Recent Advances in Lipid Nanoparticles for Cancer Vaccines: From Antigen Delivery to Personalized Immunotherapy

**DOI:** 10.3390/ijms27209023

**Published:** 2026-10-12

**Authors:** Hari Krishnareddy Rachamala, Ramcharan Singh Angom, Srujan Marepally

**Affiliations:** 1 Department of Biochemistry and Molecular Biology, Mayo Clinic College of Medicine and Sciences, Jacksonville, FL 32224, USA; angom.ramcharan@mayo.edu; 2 Centre for Stem Cell Research (CSCR) (A Unit of BRIC-InStem), Christian Medical College Campus, Bagayam, Vellore 632002, Tamil Nadu, India; srujankm@cmcvellore.ac.in

**Keywords:** lipid nanoparticles, mRNA cancer vaccines, personalized neoantigens, cancer immunotherapy, immune-targeted delivery, tumor microenvironment

## Abstract

In recent years, lipid nanoparticles (LNPs) have shown great promise as highly flexible delivery systems for cancer vaccines, which enable effective cargo delivery and allow for controlling their distribution, cell-targeting ability and immunogenicity. Innovations in ionizable lipid chemistry, biodegradable formulations, RNA engineering and LNP design resulted in improvements in RNA encapsulation, endosomal escape, delivery outside the liver, and targeting of lymphoid organs and antigen-presenting cells. All these achievements led to an expansion of strategies for developing cancer vaccines from using only mRNA to personal neoantigen vaccines, self-amplifying RNA (saRNA), circular RNA (circRNA), peptide/protein vaccines, and multi-purpose platforms that combine antigen delivery and modulation of the immune system. LNPs are also studied as tools for altering the tumor microenvironment (TME) and boosting anti-tumor immunity through local delivery of cytokines and innate immune modulators. Preliminary clinical results with personalized mRNA neoantigen vaccines show strong potential for this approach. However, inflammatory toxicity, suitability for repeated dosing, off-target delivery, difficulties with large-scale production, formulation stability, personalized production schedule and regulatory issues are still significant challenges. New biodegradable and PEG-free formulations, innovations in manufacturing and lyophilization technology, and AI-based approaches in lipid screening could help overcome some of these problems.

## 1. Introduction

Therapeutic cancer vaccines aim to induce tumor-specific adaptive immunity against tumor-associated antigens and patient-specific neoantigens. However, their efficacy remains constrained by inefficient antigen presentation, tumor heterogeneity, immune escape, an immunosuppressive tumor microenvironment (TME), and difficulties in establishing durable anti-tumor T-cell responses [1]. Recent clinical evidence from personalized RNA-based neoantigen vaccination, including studies in pancreatic ductal adenocarcinoma (PDAC), demonstrates that vaccination can generate long-lived, functional tumor-specific T-cell responses, supporting the potential of individualized cancer immunotherapy [2].

Lipid nanoparticles (LNPs) provide a versatile platform for cancer-vaccine delivery by protecting nucleic-acid cargo, facilitating cellular uptake and cytoplasmic release, and enabling control over biodistribution and immune-cell targeting. Their composition and physicochemical properties can be engineered to promote delivery to lymphoid tissues and antigen-presenting cells (APCs) while limiting off-target exposure. In addition to conventional mRNA, LNP-based platforms can accommodate self-amplifying RNA (saRNA), circular RNA (circRNA), peptides, proteins, and immunomodulatory cargo, providing opportunities to coordinate antigen delivery with immune stimulation.

Recent advances in ionizable lipid chemistry, biodegradable formulations, endosomal escape, and tissue- and cell-selective delivery are shifting LNP development from conventional nucleic-acid transport toward programmable immunotherapeutic systems. These developments are particularly relevant to therapeutic cancer vaccination, where efficient APC targeting, controlled immune activation, reduced off-target exposure, and compatibility with repeated administration are important design considerations. In parallel, advances in RNA engineering, personalized neoantigen selection, and computational approaches to lipid discovery are expanding the design space for individualized cancer vaccines.

This review examines recent advances in LNP-based cancer vaccination through a translational framework, with emphasis on LNP design and engineering, mRNA and personalized neoantigen vaccines, saRNA and circRNA platforms, immune-targeted delivery, combination immunotherapy, clinical translation, and emerging technologies. Attention is given to how LNP composition and cargo design influence immune targeting and anti-tumor responses, as well as the remaining challenges associated with safety, repeated dosing, manufacturing, stability, and personalized vaccine production.

A literature search was conducted using PubMed, Web of Science, and Scopus. Search terms included combinations of “lipid nanoparticles,” “LNP,” “cancer vaccine,” “mRNA vaccine,” “neoantigen vaccine,” “circular RNA,” “self-amplifying RNA,” and “cancer immunotherapy.” Original research articles, clinical studies, and relevant reviews addressing LNP design, vaccine delivery, immune targeting, safety, manufacturing, and clinical translation were considered. Studies unrelated to cancer vaccination, those with limited relevance to lipid-based delivery, and non-peer-reviewed publications were excluded, with emphasis placed on recent advances in LNP engineering and cancer-vaccine applications.

## 2. LNP Design and Engineering for Cancer Vaccines

The therapeutic efficacy of lipid nanoparticle (LNP)-based cancer vaccines is strongly influenced by nanoparticle composition and physicochemical properties, which govern RNA encapsulation, intracellular delivery, biodistribution, and cellular targeting [3]. Conventional mRNA-LNPs typically comprise an ionizable lipid, helper phospholipid, cholesterol, and PEG-lipid. Ionizable lipids facilitate nucleic-acid encapsulation and, following protonation in acidic endosomes, promote membrane destabilization and cytosolic RNA release [4,5]. Helper phospholipids and cholesterol contribute to particle organization and stability, whereas PEG-lipids regulate particle size, colloidal stability, and interactions with biological components. Current engineering strategies therefore focus on optimizing lipid chemistry, biodegradability, intracellular release, and tissue or immune-cell selectivity while minimizing off-target exposure and inflammatory toxicity.

### 2.1. Ionizable Lipids and Major LNP Components

Ionizable-lipid chemistry provides substantial flexibility for controlling LNP performance. Headgroup structure, linker chemistry, hydrophobic tails, branching, and apparent pKa collectively influence nanoparticle formation, intracellular trafficking, endosomal escape, and tissue tropism [6]. The large chemical space available for ionizable lipids has also motivated the integration of artificial intelligence (AI), machine learning, and high-throughput synthesis into lipid discovery.

Wang et al. combined predictive modeling and virtual screening of nearly 20 million structures with experimental validation to identify ionizable lipids with favorable apparent pKa and in vivo mRNA-delivery properties. This approach identified LQ089, which achieved delivery efficiency comparable to SM-102, and revealed structural determinants including tail length, branching, ester-linker position, and headgroup chemistry that influence lipid activity [6].

Similarly, the AI-Guided Ionizable Lipid Engineering (AGILE) platform integrated deep learning, virtual screening, and high-throughput combinatorial synthesis to identify H9, which produced approximately 7.8-fold greater intramuscular expression than MC3 with reduced liver and spleen expression. Retraining with cell-specific datasets further identified R6 as a lipid with preferential macrophage delivery [7].

Beyond computational discovery, individual structural elements can substantially alter LNP behavior. Somu Naidu et al. demonstrated that changes in linker chemistry—including ester, carbonate, amide, reverse-amide, and urea linkages—altered mRNA biodistribution, with selected amide- and urea-containing lipids showing enhanced stability and preferential pulmonary transfection [8].

Headgroup chemistry provides another level of control. GCP-containing ionizable lipids led to the cholesterol-free GL5-3 formulation, which redirected mRNA expression from the liver toward the spleen. Its pH-responsive behavior also promoted membrane interactions under acidic endosomal conditions, supporting cytoplasmic RNA delivery [9].

These findings indicate that LNP tropism cannot be assigned to a single structural feature. Rather, headgroup chemistry, linker architecture, hydrophobic domains, and overall formulation act together to determine delivery efficiency and tissue or cellular selectivity. For cancer vaccination, this shifts the design objective from maximizing total mRNA expression toward achieving efficient delivery to immunologically relevant tissues and cell populations.

### 2.2. Degradable Ionizable Lipids and Next-Generation LNP Formulations

Persistent or nonspecific accumulation of synthetic lipids can contribute to inflammation and systemic toxicity, making biodegradability particularly important for cancer vaccines that may require repeated administration [10]. Next-generation formulations therefore aim to combine efficient delivery with rapid lipid clearance and reduced off-target exposure.

Rudra et al. developed AMG1541, a rapidly degradable ionizable lipid with an intact-lipid half-life of approximately 3 h. Compared with SM-102, AMG1541 reduced hepatic expression while increasing delivery to APCs at the injection site and draining lymph nodes. Although evaluated in an influenza mRNA-vaccine model, its combination of rapid clearance, APC delivery, and activity at low RNA doses illustrates properties relevant to repeated cancer vaccination [11].

Stimulus-responsive degradation can additionally couple intracellular delivery with controlled lipid clearance. Chen et al. developed crown-like biodegradable ionizable lipids containing metal-coordinating amino headgroups and disulfide-bridged ester linkers. Zinc coordination with 9C-SCC-10 redirected mRNA expression from the liver toward the lung, demonstrating that biodegradability and organ-selective delivery can be incorporated within the same lipid architecture [12].

Next-generation formulations can also reduce reliance on conventional helper components. Cholesterol- and PEG-free ThrCo LNPs decreased hepatic accumulation by approximately 70% and increased splenic mRNA translation by 4.5-fold relative to MC3 while maintaining delivery efficiency after repeated administration. Preferential delivery to splenic dendritic cells and macrophages enhanced CD8^+^ T-cell responses, and vaccination in the B16F10-OVA melanoma model inhibited primary tumor growth, metastasis, and recurrence while promoting immune memory and survival [13].

Delivery efficiency must also be balanced with endosomal escape and lipid exposure. Excessive membrane disruption can promote inflammation; notably, 4A3-SC8 maintained efficient mRNA expression with limited membrane damage, supporting controlled rather than maximal endosomal destabilization [14]. Alternatively, Mn^2+^-mediated mRNA condensation (L@Mn-mRNA) increased RNA loading and dendritic-cell uptake while reducing the lipid dose and anti-PEG antibody production [15]. Together, these findings highlight the importance of coordinating lipid biodegradability, RNA loading, and intracellular release to improve the therapeutic window of LNP cancer vaccines (Figure 1).

Together, these approaches demonstrate that biodegradability should be considered alongside tissue selectivity, immune-cell delivery, and repeat-dose compatibility rather than as an isolated design objective. Representative next-generation LNP engineering strategies are summarized in Table 1.

## 3. LNP-Based Cancer Vaccine Strategies

LNPs support multiple cancer-vaccine modalities, including conventional mRNA, personalized neoantigen-encoding RNA, saRNA, circRNA, peptides, proteins, and immunomodulatory cargo. These approaches differ in antigen-expression kinetics, cargo requirements, immunogenicity, and capacity for combination with immune modulators. This section compares their major design principles and therapeutic applications [17].

### 3.1. mRNA and Personalized Neoantigen Vaccines

mRNA vaccines are attractive for cancer immunotherapy because they enable transient expression of tumor-associated or tumor-specific antigens without genomic integration. Following delivery to APCs, translated antigens can be processed through MHC class I and II pathways to activate CD8^+^ and CD4^+^ T-cell responses [18,19]. The efficacy of mRNA-LNP vaccines depends not only on antigen expression but also on where and how the RNA is delivered. Large, low-PEG LNPs enhanced dendritic-cell targeting and generated cDC1-dependent cytotoxic T-cell responses against HPV E6/E7 antigens at an mRNA dose of 1 μg [19]. Similarly, esomeprazole-containing formulations enhanced cytoplasmic mRNA delivery, dendritic-cell activation, antigen-specific CD4^+^ and CD8^+^ T-cell responses, and tumor control in the B16-OVA model [20]. Spatial and temporal control of antigen expression provides an additional means of improving vaccination. Muscle-selective zwitterionic LNPs promoted localized antigen expression and cDC1-mediated anti-tumor immunity [21], whereas an injectable LiNx mRNA-LNP depot prolonged antigen availability and supported durable CD8^+^ T-cell memory after a single administration [22]. Inhaled mRNA-LNPs similarly generated local antigen expression and CD8^+^ T-cell immunity that protected against pulmonary metastases [23]. LNPs can also coordinate antigen expression with immune stimulation. Co-delivery of antigen-encoding mRNA and the STING agonist cGAMP using mannose-targeted nanovaccines enhanced antigen-specific T-cell responses and improved anti-tumor activity in combination with PD-1/PD-L1 blockade [24]. These findings indicate that mRNA-LNP efficacy is determined by the integration of antigen expression, APC targeting, intracellular delivery, and immune activation rather than by RNA expression alone.

Personalized neoantigen vaccines extend this approach by encoding tumor-specific somatic mutations absent from normal tissues. Their development involves tumor and normal-tissue sequencing, identification of somatic mutations, HLA typing, computational neoantigen prioritization, and incorporation of selected epitopes into vaccine formulations. Multiepitope vaccination can broaden tumor-specific immunity and potentially reduce immune escape associated with intratumoral heterogeneity [25,26]. Personalized vaccination has generated neoantigen-specific CD4^+^ and CD8^+^ T-cell responses in glioblastoma and robust T-cell responses in high-risk clear-cell renal cell carcinoma, demonstrating the feasibility of identifying therapeutically relevant neoantigens across tumors with different mutational burdens [25,26]. However, clinical translation remains constrained by neoantigen-prediction accuracy, tumor and HLA heterogeneity, manufacturing complexity, cost, and production time. Thus, the development of personalized mRNA-LNP vaccines requires integration of accurate antigen selection with efficient delivery and rapid, scalable manufacturing.

### 3.2. Self-Amplifying and Circular RNA Vaccines

The magnitude and duration of antigen expression are important determinants of vaccine performance. saRNA and circRNA offer alternative RNA architectures that can prolong antigen availability or reduce the amount of RNA required for vaccination [27].

circRNA is resistant to exonucleolytic degradation because of its covalently closed structure. LNP-delivered circRNAs encoding tumor-associated antigens, neoantigens, and oncoviral antigens demonstrated enhanced RNA stability, lymph-node and APC accumulation, durable CD4^+^ and CD8^+^ T-cell responses, and tumor-growth suppression in multiple syngeneic models; therapeutic activity was further enhanced by immune-checkpoint blockade [28].

Importantly, LNP formulations producing the greatest circRNA expression did not necessarily generate the strongest immune responses, indicating that transfection efficiency alone is an inadequate surrogate for vaccine immunogenicity [29]. This suggests that transfection efficiency is not enough to predict the immunogenicity of the vaccine.

saRNA instead prolongs antigen expression through intracellular RNA amplification. LNP-delivered Venezuelan equine encephalitis virus-derived saRNA produced more sustained antigen expression than conventional mRNA-LNPs and elicited robust CD4^+^ and CD8^+^ T-cell responses [27]. ALC-0315-based LNPs similarly supported strong T-cell responses at low saRNA doses, with membrane-anchored antigen outperforming secreted antigen [30]. Although these studies included infectious-disease antigens, they establish design principles relevant to cancer-vaccine development.

Thus, saRNA and circRNA address antigen persistence through distinct mechanisms: intracellular amplification and enhanced molecular stability, respectively. Their relative advantages for cancer vaccination will require direct evaluation across comparable formulations, doses, administration routes, and tumor models.

### 3.3. Peptide/Protein and Combination Vaccine Platforms

Peptide- and protein-based vaccines provide defined antigen composition and relatively straightforward manufacturing but are limited by antigen stability, lymphatic trafficking, APC uptake, and cross-presentation. Nanoparticle delivery can address these limitations while enabling co-delivery of antigens and immunostimulatory agents.

For example, incorporation of glutamate-modified peptide epitopes and a TLR7/8 agonist into ionizable LNPs enhanced APC uptake, antigen-specific CD8^+^ T-cell responses, and protective anti-tumor immunity [31]. Similarly, pH-sensitive nanoparticles co-delivering peptide neoantigens with the STING agonist cGAMP and TLR4 agonist MPLA enhanced dendritic-cell activation, cross-presentation, lymph-node accumulation, tumor control, and responsiveness to immune-checkpoint blockade [32].

The organization and selection of immunostimulatory cargo can further influence vaccine activity. Screening of lipid-phase adjuvants identified formulations that enhanced neoantigen-specific CD8^+^ T-cell responses and therapeutic activity [33]. A svg3, an oligonucleotide-based cGAS agonist delivered with peptide or mRNA antigens, promoted draining lymph-node and APC targeting, enhanced antigen presentation and CD8^+^ T-cell responses, and improved checkpoint-blockade efficacy [34].

Spatial organization of the TLR3 agonist poly(I:C) within the nanoparticle core and peptide antigen at the surface likewise generated potent antigen-specific CD8^+^ T-cell responses and therapeutic activity [35].

These approaches illustrate a central advantage of nanoparticle-based combination vaccines: antigen delivery and innate immune activation can be coordinated within the same formulation. However, differences in antigen identity, adjuvant, formulation, dose, and tumor model currently limit direct comparisons. Representative vaccine strategies and their major immunological and therapeutic outcomes are summarized in Table 2.

## 4. Tissue- and Immune Cell-Selective LNP Delivery for Cancer Vaccination

The efficacy of LNP-based cancer vaccines depends not only on efficient cargo delivery but also on directing that cargo to tissues and cell populations capable of initiating productive anti-tumor immunity. Conventional LNPs frequently exhibit hepatic-biased or broad biodistribution, whereas cancer vaccination often requires delivery to lymphoid tissues, tumors, APCs, or defined immune-cell populations. Next-generation formulations therefore use lipid composition, particle architecture, administration route, and surface functionalization to achieve tissue- and cell-selective delivery while minimizing off-target exposure [37]. This section considers targeting at three interconnected levels: tissue-selective biodistribution, immune-cell-specific delivery, and the resulting antigen presentation and T-cell immunity (Figure 2).

### 4.1. Tissue-Selective and Extrahepatic Delivery

Lymph nodes (LNs) provide specialized microenvironments in which APCs acquire vaccine-derived antigens and prime tumor-reactive T-cells. Preferential delivery to draining LNs (dLNs) can therefore increase antigen availability and APC activation while limiting unnecessary systemic exposure. Kubara et al. identified L17-F05 through screening in primary human APCs. Following subcutaneous administration, L17-F05 delivered tumor-antigen-encoding mRNA to dLNs, where conventional dendritic cells mediated antigen presentation and lymph-node macrophages promoted STING-dependent type I interferon signaling. Vaccination with gp100- and Trp2-encoding mRNAs suppressed B16F10 melanoma growth and prolonged survival [36]. Similarly, 113-O12B LNPs preferentially mediated mRNA expression in LNs and enhanced antigen-specific CD8^+^ T-cell responses and anti-tumor activity, with anti-PD-1 treatment further promoting durable tumor control and immunological memory [38]. Aromatic bio-reducible ionizable lipids containing disulfide linkages also promoted LN-selective expression while reducing hepatic transfection and generating antigen-specific and effector-memory T-cell responses [18]. These findings indicate that successful LN targeting should be assessed by productive APC delivery and subsequent T-cell immunity rather than nanoparticle accumulation alone.

More broadly, redirecting LNP biodistribution beyond the liver is an important objective for cancer vaccination. Biodistribution is influenced by lipid chemistry, particle architecture, and administration route, with intravenous, intramuscular, subcutaneous, intratumoral, and inhaled delivery producing distinct patterns of tissue exposure. Changes in ionizable-lipid linker chemistry can substantially alter organ tropism, with selected amide- and urea-containing lipids preferentially mediating pulmonary transfection [8]. LNPs enriched in bilayer-forming sphingomyelin and cholesterol have also demonstrated broader extrahepatic distribution, including lymph nodes and macrophage-rich regions of the spleen [5]. In addition, cholesterol- and PEG-free ThrCo LNPs reduced hepatic accumulation while increasing splenic mRNA expression and productive delivery to splenic APCs, resulting in enhanced antigen-specific CD8^+^ T-cell responses and anti-tumor activity [13]. These approaches illustrate how LNP composition and administration can be used to shift delivery from the liver toward tissues relevant to cancer vaccination.

### 4.2. APC and Immune-Cell-Selective Delivery

Direct targeting of APCs provides an additional level of specificity. Dendritic cells are particularly important because they coordinate antigen processing, MHC-mediated presentation, costimulation, and cytokine signaling required for tumor-specific T-cell priming [31].

APC selectivity can be achieved through both physicochemical and receptor-mediated strategies. Large, low-PEG LNPs preferentially delivered mRNA to dendritic cells and generated strong effector and memory T-cell responses, demonstrating that particle size and PEG density can influence APC tropism without additional targeting ligands [18]. Receptor-mediated approaches provide greater cellular specificity. Mannose-modified, pH-responsive LNPs enhanced dendritic-cell targeting and intracellular mRNA release, resulting in increased antigen expression, DC activation, and anti-tumor immunity [39]. DEC-205-functionalized LNPs similarly promoted delivery to lymph nodes and tumors, while co-delivery of antigen-encoding mRNA and Toll-like receptor agonists enhanced antigen presentation and CD8^+^ T-cell responses [17].

These approaches demonstrate that APC targeting requires more than cellular uptake. Productive vaccine delivery depends on reaching functionally relevant APC subsets, achieving cytoplasmic cargo release, and supporting antigen presentation while limiting off-target transfection.

### 4.3. Antigen Presentation and T-Cell Immunity

Tissue and cellular targeting provide therapeutic benefit only when delivered antigens are efficiently processed and presented to generate functional tumor-specific T-cell responses. Recent mechanistic studies indicate that mRNA-LNP vaccination can engage multiple dendritic-cell subsets and antigen-presentation pathways rather than relying exclusively on conventional cDC1-mediated cross-presentation.

Muro et al. found that mRNA-LNP vaccination generated substantially greater antigen-specific CD8^+^ T-cell responses than protein antigen delivered with conventional immunostimulants. Following intramuscular administration, mRNA-LNPs were acquired predominantly by migratory cDC2s in dLNs and supported prolonged MHC-I antigen presentation. CD8^+^ T-cell priming depended on migratory dendritic cells but was independent of the conventional cross-presentation pathway [40].

Consistent with this observation, Jo et al. showed that both cDC1 and cDC2 populations can contribute to mRNA-LNP-induced CD8^+^ T-cell priming and that T-cells generated through different DC populations retain anti-tumor activity and memory potential [17]. LNPs can further coordinate antigen presentation with localized T-cell stimulation. The vaccine-initiated selective T-cell modulation (VISIT) strategy delivered mRNAs to splenic dendritic cells, enabling simultaneous tumor-antigen presentation and expression of membrane-bound IL-15/IL-15Rα complexes. This spatial coordination selectively expanded antigen-specific cytotoxic T-cells while limiting nonspecific T-cell activation and enhanced anti-tumor immunity [41].

Thus, the objective of immune-targeted LNP vaccination extends beyond maximizing antigen expression. Effective platforms must coordinate the anatomical location, cellular source, and duration of antigen presentation with appropriate costimulatory and immune-activating signals to generate selective and durable tumor-specific T-cell immunity.

## 5. LNPs for Immune Modulation and Combination Therapy

In addition to simple antigen delivery, there is an ever-growing trend in developing lipid nanoparticle (LNP)-based platforms with the potential to actively remodel the tumor-associated immune system and reprogram the tumor microenvironment. LNPs’ capacity to carry multiple therapeutic cargos, including antigens, innate immune agonists, cytokines and others, allows for the coordinated activation of innate and adaptive immune responses. LNP-based vaccines can be administered in combination with immune-checkpoint inhibitors to improve vaccine-induced T-cell responses and alleviate tumor-associated immunosuppression. Collectively, these approaches will allow the transformation of LNPs from merely passive vaccine vehicles into active immunotherapeutic platforms promoting tumor-specific immunity and remodeling the immune context [24,42].

### 5.1. LNP-Mediated Immune Modulation and Co-Delivery

LNPs can coordinate antigen delivery with innate immune activation, providing a strategy to enhance antigen presentation while limiting systemic exposure to potent immunomodulators. Co-delivery of peptide antigens with TLR7/8 agonists enhanced lymphatic transport, APC activation, antigen-specific CD8^+^ T-cell responses, and anti-tumor immunity [43]. Similarly, combinations of lipid-based adjuvants targeting TLR and inflammasome pathways improved neoantigen-specific T-cell responses [33], while co-delivery of peptide or mRNA antigens with the cGAS agonist Svg3 promoted lymph-node targeting, type I interferon signaling, antigen presentation, and therapeutic activity [34]. Spatial organization of antigen and TLR3 agonists within nanoparticles can further improve immune activation while reducing adjuvant-associated toxicity [44]. These findings demonstrate that the identity, localization, and organization of immunostimulatory cargo are important determinants of vaccine activity.

Collectively, these examples show that LNPs can act as integrated immunological platforms, coordinating antigen delivery with TLR, inflammasome and cGAS-STING pathway activation. However, the choice of pathway, doses, spatial organization and timing of activation are important because excessive innate activation increases inflammation and toxicity and may interfere with productive antigen delivery and expression in the context of mRNA vaccines. Future LNP-based vaccines should consider this aspect along with rational antigen and adjuvant choice.

LNPs can also deliver innate immune agonists and cytokine-encoding mRNAs to remodel the tumor microenvironment. Co-delivery of IL-33 mRNA with the STING agonist c-di-AMP enhanced innate immune activation and immune-cell infiltration [45], whereas LNP delivery of TLR3 agonists promoted dendritic-cell activation and antigen-specific T-cell responses, with activity strongly influenced by LNP composition and biodistribution. Cytokine-based approaches provide complementary immune modulation; intratumoral delivery of IL-21, IL-7, and 4-1BBL mRNAs increased tumor-infiltrating CD8^+^ T-cells and supported anti-tumor immunity and immune memory [42]. Collectively, these studies indicate that LNPs can integrate antigen delivery, innate immune activation, and cytokine signaling, although dose and localization must be carefully controlled to avoid excessive inflammation and systemic toxicity.

### 5.2. Combination with Immune-Checkpoint Inhibitors

Immune-checkpoint inhibitors (ICIs), mainly anti-PD-1/PD-L1 antibodies, have revolutionized cancer immunotherapy; however, the efficiency of these therapies depends on the existence of tumor-reactive T-cells and a permissive tumor immune microenvironment. ‘Immunologically cold’ tumors with poor antigen presentation, low T-cell infiltration and strong inhibitory signaling rarely respond to checkpoint blockade. LNP-based cancer vaccines represent a complementary approach to ICIs, promoting antigen presentation, expansion of tumor-specific T-cells and immune-cell infiltration into tumors [46].

Jin et al. demonstrated this concept by developing mannose-modified targeted lipid nano-vaccines co-encapsulating antigen-encoding mRNA and the STING agonist cGAMP. Antigen-encoding mRNA delivery and APC targeting allowed for enhanced antigen presentation and innate immune activation, while combination with anti-PD-L1 treatment resulted in greater anti-tumor activity than each treatment alone and immune memory generation [24].

Evidence supporting the general hypothesis of RNA vaccination being capable of creating a T-cell-inflamed TME through an alternative lipid nanoparticle platform is available as well. Fournier et al. have utilized nanostructured lipid carriers (NLCs), which are different from traditional ionizable-lipid LNPs for delivering mRNA of ovalbumin (OVA). Enhanced dendritic cell (DC) activation, antigen-specific T-cell response, intratumor expression of CXCL9, CXCL10, and CXCL11, as well as infiltration of immune cells, have been observed with NLC-based mRNA vaccine [46]. The treatment became even more effective with the use of anti-PD-1 treatment. Thus, this study provides additional evidence that RNA vaccination through a lipid platform can create a T-cell-inflamed microenvironment that is favorable for checkpoint inhibition.

Zhang et al. have also demonstrated that a combination of LNP-delivered small circRNA vaccines with immune-checkpoint inhibition can enhance the anti-tumor activity of LNP vaccines. Modification-free circRNAs encoding tumor-associated antigens, neoantigens and oncoviral antigens provided prolonged antigen expression and generation of persistent CD4^+^ and CD8^+^ T-cell responses. LNP-delivered vaccination increased immune-cell infiltration and immunosuppression, while combination with checkpoint inhibition increased therapeutic anti-tumor activity in poorly immunogenic tumors, including BRAF^V600E^ melanoma, resistant to checkpoint inhibition [28]. Finally, Yang et al. have demonstrated the combination of LNP-delivered OVA mRNA and PD-L1-targeting siRNA to generate dual-engineered dendritic-cell vaccines. This approach combines antigen presentation and elimination of PD-L1-mediated inhibitory signaling. Additional systemic anti-PD-L1 treatment increased therapeutic anti-tumor activity and immune memory [42].

Collectively, these examples provide a strong biological rationale for combining LNP-based vaccination with immune-checkpoint inhibition. Vaccination generates or expands tumor-specific T-cell responses and allows for immune-cell infiltration into tumors, while PD-1/PD-L1 blockade alleviates inhibitory signaling, blocking vaccine-induced effector cells. Combination of LNP-based cancer vaccines and checkpoint inhibition may be especially useful for tumors with low T-cell infiltration or resistance to checkpoint inhibitors. Nevertheless, it is important not to assume synergistic activity of LNP vaccines and checkpoint inhibitors without appropriate analysis of drug interaction. Future studies need to evaluate optimal sequence and dosing, predictive biomarkers and clinical translation of preclinical benefits.

### 5.3. Remodeling the Tumor Microenvironment

Remodeling of tumor TME represents an important goal for successful cancer vaccination, as even tumor-reactive T-cells may not be able to infiltrate and act due to immunosuppressive TAMs, regulatory immune populations, cytokine network, dysfunctional antigen presentation and T-cell exhaustion. LNPs represent a versatile tool for overcoming such barriers by delivering siRNAs, mRNAs, innate immune agonists, and other immunomodulatory cargos to tumors and stromal compartments [47].

Targeting immunosuppressive tumor-associated macrophages (TAMs) is an attractive approach because some specific macrophage phenotypes can promote tumor progression and inhibit cytotoxic T-cell activity. Gao et al. have developed LNPs carrying siRNA against Forkhead box M1 (FOXM1) in breast cancer. Inhibition of FOXM1 resulted in an increase in Krüppel-like factor 15 (KLF15), a decrease in M2-like macrophage infiltration and an increase in more inflammatory macrophages. These changes inhibited tumor-cell proliferation and invasion, and enhanced sensitivity to immune-checkpoint inhibition [48].

Selectivity and specificity in TAM targeting were achieved by Liang et al. Identifying SPP1+ macrophages as an immunosuppressive population associated with poor immunotherapy responses in non-small-cell lung cancer, the authors developed mannose-modified SORT LNPs for lung-restricted delivery of SPP1-targeting siRNA and IFN-γ mRNA. This combination resulted in suppression of the SPP1-mediated tumor-promoting program while simultaneously providing a pro-inflammatory cytokine signal, reprogramming macrophages toward a more tumoricidal phenotype. Combination with immune-checkpoint inhibition additionally increased anti-tumor activity in orthotopic and pulmonary-metastasis models [47].

Another promising direction is a direct in vivo engineering of TAMs with synthetic tumor recognition functions. Xu et al. have developed fluorinated ionizable LNPs for TAM-specific mRNA delivery. A1F5C5 has been chosen as the most efficient formulation among tested LNPs for macrophage reprogramming. LNP delivery of mRNA encoding a PSMA-targeted chimeric antigen receptor (CAR) resulted in the generation of CAR macrophages in vivo after systemic delivery. This approach reduced populations of immunosuppressive macrophages and increased granzyme B and perforin expression in intratumoral CD8^+^ T-cells. Combination with anti-PD-L1 provided complete and durable tumor regression in the MC38-hPSMA model [49].

Collectively, these examples illustrate the evolution of LNP-based cancer immunotherapy from passive cargo delivery to active reprogramming of the tumor microenvironment. Targeting of TAMs and inhibition of tumor-promoting programs can change immune state, increase inflammatory and tumoricidal functions, and recreate the conditions necessary for cytotoxic T-cell activity. The capability to combine gene silencing with cytokine expression and in vivo macrophage engineering with synthetic receptors provides new possibilities for in vivo reprogramming of immune cells.

However, TAM reprogramming requires special care due to the high heterogeneity of macrophage states, which cannot always be described by simple binary M1/M2 classification. Uncontrolled inflammation may cause tissue damage and systemic toxicity, while incomplete targeting may affect non-targeted cells and tissues. Future LNP-based platforms should allow for precise targeting of immunosuppressive populations, transient and controllable cargo expression, and integration with tumor-specific vaccination and checkpoint inhibition. Representative immune-targeted and immunomodulatory LNP strategies and their mechanisms of anti-tumor immune activation are summarized in Table 3. Together, these strategies illustrate how multifunctional LNPs can integrate antigen delivery, innate immune activation, cytokine signaling, immune-cell reprogramming, and checkpoint blockade to convert an immunosuppressive “cold” TME into an immune-activated “hot” TME (Figure 3).

## 6. Preclinical and Clinical Advances

Recent developments in the field of lipid nanoparticle (LNP) engineering have yielded positive results concerning preclinical and clinical testing of cancer vaccination, including improvements in antigen delivery, APC activation, tumor-specific T-cell immunity, immune memory formation, and anti-tumor effect. Personalized mRNA vaccines also achieved important milestones in clinical testing, demonstrating feasibility, safety, persistence of neoantigen-specific immunity, and clinical benefit in certain settings [52].

### 6.1. Major Advances Across Cancer Models

The current preclinical research is focused on integrating antigen delivery with innate immunity activation and modulating the tumor microenvironment. For example, the nAg-MRDE/Mn nanovaccine combined personalized neoantigens and activation of the cGAS–STING pathway mediated by Mn^2+^, leading to improved antigen cross-presentation, DC maturation, NK cell activation, CD8^+^ T-cell responses, and anti-tumor activity [53]. Similarly, the biomimetic AECM@PC7A system integrated tumor-derived antigens and STING activation, leading to DC maturation and multi-epitope CD8^+^ T-cell responses [54].

There are also efforts directed at using spatial control and pre-existing immunity. The LiNx nanofiber–hydrogel depot delivered mRNA-LNP locally and provided improved antigen presentation and long-lasting memory of CD8^+^ T-cells, resulting in powerful anti-tumor activity with single vaccination [22]. In another approach, AA2/AA15V LNPs redirected pre-existing spike-specific immunity towards tumor cells, allowing CD8^+^ T-cell recognition and tumor suppression in models of melanoma and lymphoma [36]. Overall, these results indicate that successful vaccination against cancer should incorporate coordinated antigen delivery and immune activation along with TME modulation.

### 6.2. Clinical Development of mRNA-LNP and Personalized Neoantigen Vaccines

Personalized cancer vaccines have progressed from early feasibility studies to randomized clinical testing. In a Phase I study, autogene cevumeran encoding up to 20 personalized neoantigens induced neoantigen-specific CD4^+^ and/or CD8^+^ T-cell responses in 71% of evaluable patients with advanced solid tumors [55]. In resected high-risk melanoma, intismeran autogene (mRNA-4157/V940), encoding up to 34 personalized neoantigens, was evaluated with pembrolizumab in the randomized Phase IIb KEYNOTE-942 trial and reduced the risk of recurrence or death compared with pembrolizumab alone, with longer follow-up demonstrating recurrence-free and distant metastasis-free survival benefits [52,56].

Personalized RNA vaccination has also demonstrated durable immunogenicity in pancreatic ductal adenocarcinoma (PDAC). Autogene cevumeran induced vaccine-specific T-cell responses in 8 of 16 vaccinated patients, with delayed recurrence among responders, while long-term follow-up demonstrated persistent vaccine-specific CD8^+^ T-cell clones with effector and memory phenotypes and prolonged recurrence-free survival [2,57].

Clinical experience with other personalized neoantigen vaccine platforms provides additional insights relevant to LNP-based vaccination. Personalized multiepitope vaccination in high-risk clear-cell renal cell carcinoma induced vaccine-specific T-cell responses in all nine patients, with vaccine-related clonotypes persisting for up to three years, although validation in larger randomized cohorts is required [25]. Similarly, the high-capacity DNA vaccine GNOS-PV02, encoding up to 40 personalized neoantigens and administered with an IL-12 plasmid and pembrolizumab in advanced hepatocellular carcinoma, induced neoantigen-specific CD4^+^ and CD8^+^ responses and intratumoral trafficking of vaccine-associated T-cells [58]. Personalized vaccination has also demonstrated immunogenicity in glioblastoma using GNOS-PV01 containing up to 40 personalized neoantigens with an IL-12 plasmid adjuvant [26]. Although these platforms are not LNP-based, they provide useful information regarding antigen breadth, tumor heterogeneity, and persistence of personalized immune responses.

Collectively, these clinical studies demonstrate the feasibility of generating broad and durable neoantigen-specific immunity but also highlight important limitations. Neoantigen-prediction accuracy, tumor and HLA heterogeneity, manufacturing time and cost, antigen loss, and interpatient variability in immune competence remain significant barriers. Moreover, induction of vaccine-specific T-cell responses does not necessarily translate into objective clinical responses. Future development therefore requires adequately powered randomized trials, improved neoantigen selection, shorter manufacturing timelines, scalable multivalent delivery, and systematic evaluation of long-term safety and repeat-dose feasibility (Table 4).

## 7. Challenges and Emerging Technologies

Despite substantial advances in LNP-based cancer vaccines, clinical translation remains limited by inflammatory toxicity, repeat-dose compatibility, off-target delivery, manufacturing reproducibility, formulation stability, and the complexity of personalized vaccine production. Controlled endosomal escape and rapidly biodegradable or PEG-free formulations may improve safety by reducing membrane injury, hepatic exposure, and anti-carrier effects while maintaining delivery to immunologically relevant tissues [11,14,35,59].

Manufacturing and stability are additional barriers, particularly for personalized vaccines requiring rapid production of small batches. Scalable mixing and downstream processing, quality-by-design approaches, and automated process monitoring can improve reproducibility, whereas lyophilization may enhance storage stability provided that freezing, reconstitution, and biological activity are carefully controlled [35,59,60,61].

Personalized vaccines introduce further logistical and regulatory challenges because each product incorporates patient-specific antigen sequences. Clinical implementation requires integration of tissue acquisition, sequencing, neoantigen selection, mRNA production, LNP formulation, quality control, and release within clinically relevant timelines. Automation of sequencing and bioinformatics, modular GMP manufacturing, rapid release testing, and reliable chain-of-identity procedures will therefore be important for scalable personalized vaccination [55,62,63].

Emerging technologies may address several of these challenges simultaneously. AI-guided lipid discovery, machine learning, high-throughput synthesis, biodegradable lipid chemistry, and predictive formulation modeling can accelerate optimization of delivery efficiency, tissue selectivity, safety, stability, and repeat-dose performance [6,7,64]. Ultimately, successful translation of LNP-based cancer vaccines will require balancing delivery potency with safety, manufacturability, stability, and the practical demands of individualized treatment.

## 8. Future Perspectives and Conclusions

LNP-based cancer vaccines are evolving from conventional delivery systems into programmable immunotherapeutic platforms capable of coordinating antigen delivery, immune-cell targeting, and immune modulation. Advances in ionizable-lipid chemistry, RNA engineering, biodegradable formulations, and tissue-selective delivery have expanded their potential for generating tumor-specific immunity. However, clinical translation will depend less on maximizing RNA expression alone than on achieving appropriate biodistribution, productive intracellular delivery, and durable anti-tumor immunity with acceptable safety.

A major priority is improving tissue and cell selectivity while maintaining compatibility with repeated administration. Future LNPs should minimize unnecessary hepatic and systemic exposure while enhancing delivery to lymphoid tissues, APCs, and tumors. This will require coordinated optimization of lipid biodegradability, endosomal escape, and dose efficiency, because improvements in intracellular delivery must be balanced against membrane damage and inflammatory toxicity [6,13].

Personalized cancer vaccination represents an important translational opportunity but introduces additional challenges in neoantigen selection, manufacturing speed, and scalability. Integration of tumor sequencing, HLA typing, computational neoantigen prioritization, automated bioinformatics, and modular manufacturing could shorten the interval between tumor sampling and vaccination. AI-guided lipid discovery and high-throughput screening may further accelerate the identification of formulations optimized simultaneously for delivery efficiency, tissue selectivity, biodegradability, and safety [65].

Clinical implementation will also require reproducible and scalable manufacturing, standardized quality control, improved formulation stability, and efficient production workflows. These requirements are particularly important for individualized vaccines, for which manufacturing timelines, batch consistency, storage, and rapid release testing directly influence clinical feasibility [59].

Ultimately, the therapeutic value of LNP cancer vaccines will depend on integrating delivery engineering with tumor biology and patient-specific immune context. Rational combinations with checkpoint blockade or other immunomodulatory therapies may further improve efficacy, but their selection should be guided by mechanistic and clinical evidence rather than increasing therapeutic complexity alone. Continued progress in safety, immune-cell selectivity, repeat-dose compatibility, scalable manufacturing, and clinical validation will determine whether the versatility demonstrated by LNP-based cancer vaccines can be translated into consistent and durable clinical benefit.

## Figures and Tables

**Figure 1:**
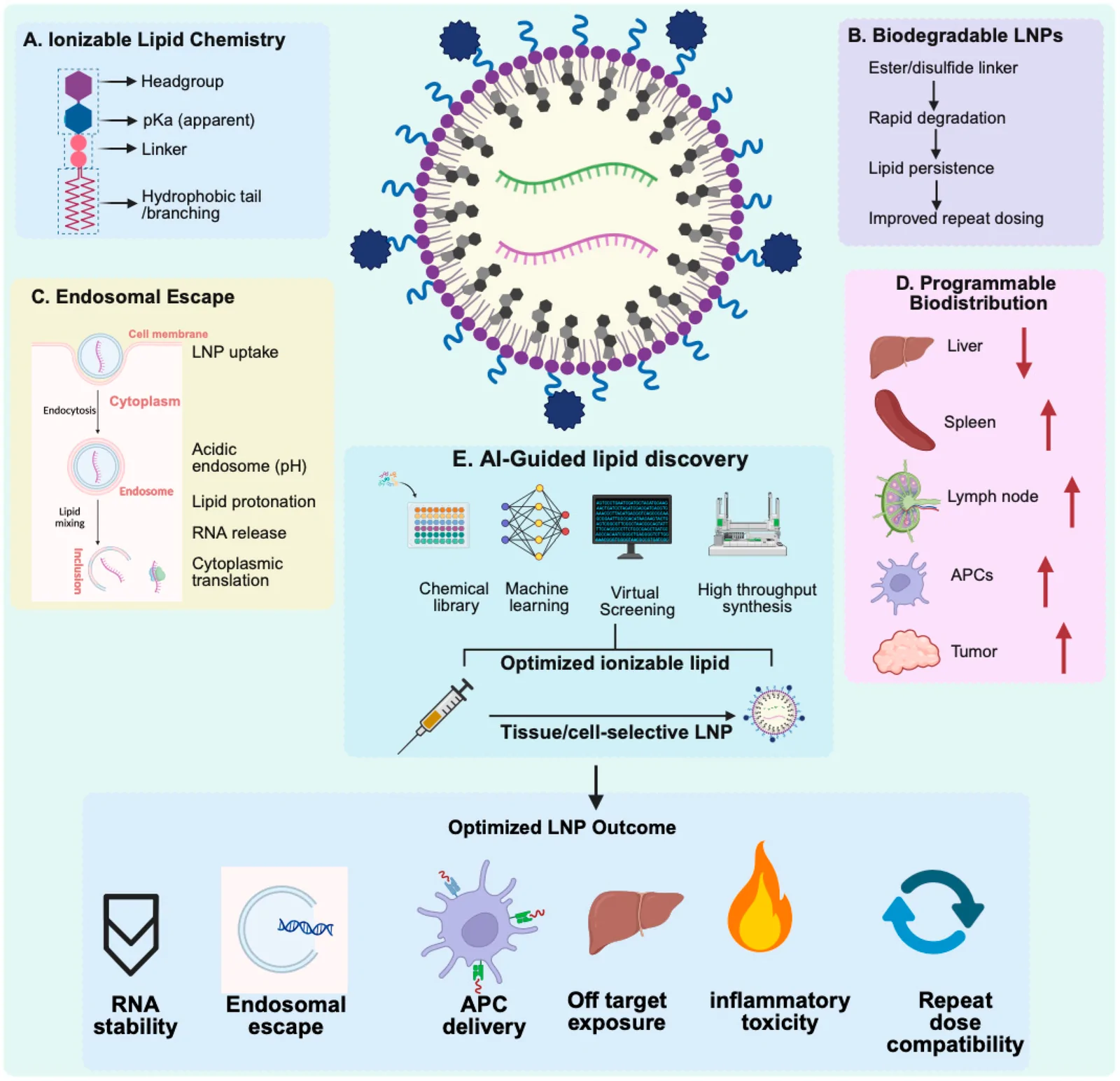
Principles of engineering that drive the design of future LNPs used in cancer vaccines. Major approaches include: (**A**) chemical modifications of ionizable lipids to increase efficacy of LNPs; (**B**) use of biodegradable linkers to decrease accumulation of lipids and allow for repeated doses; (**C**) optimization of endosome escape for more effective cytoplasmic RNA delivery; (**D**) preferential targeting of lymphoid organs, APCs, and tumor tissue with minimal exposure to the liver; and (**E**) the use of AI, virtual screening, and high-throughput synthesis to obtain optimized, tissue-specific lipids.

**Figure 2:**
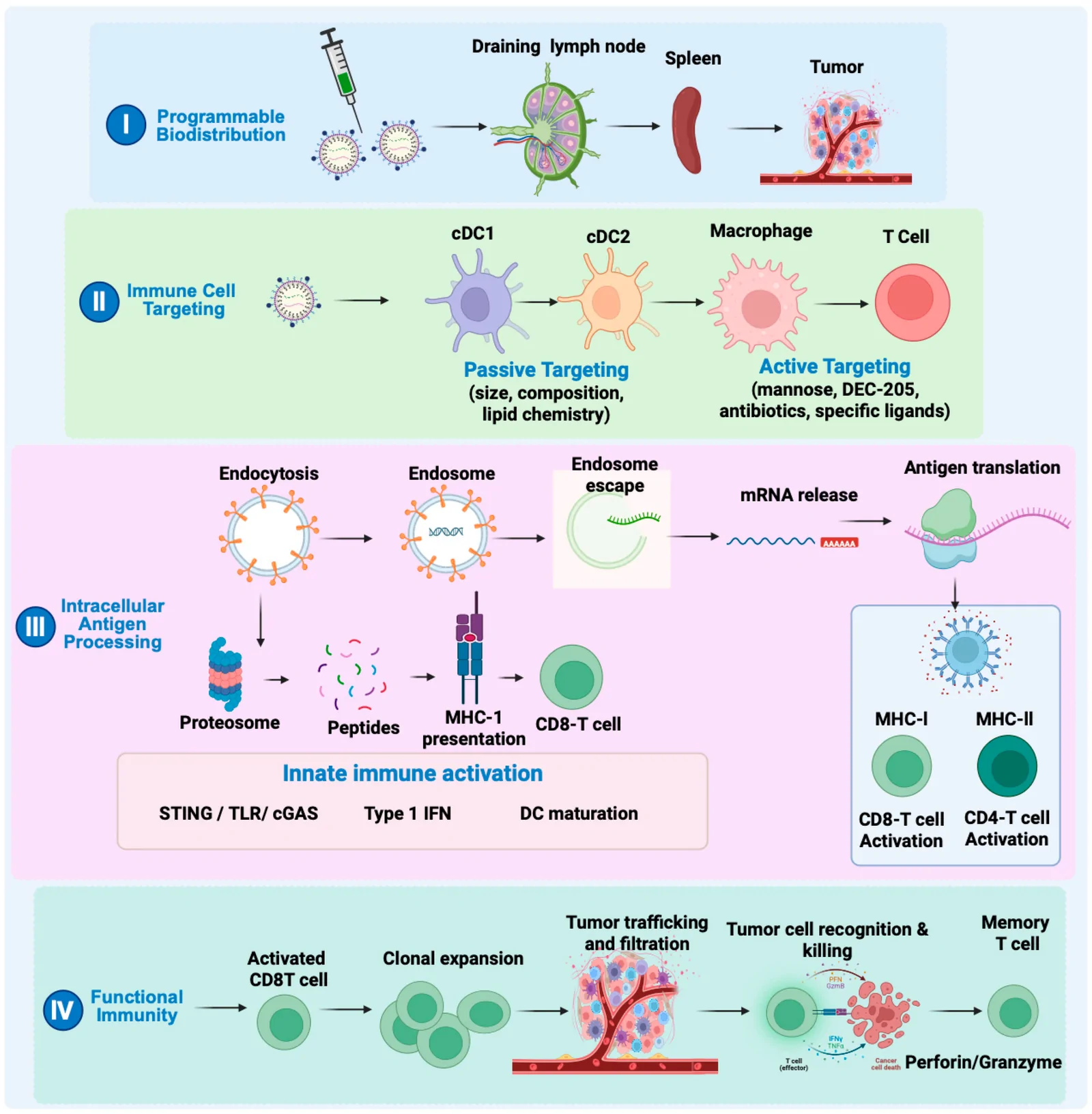
Immune-targeted delivery and antigen-processing pathways of LNP-based cancer vaccines. This figure shows the mechanism steps involved in the use of LNP for cancer immunization: (**I**) targeted delivery of the LNPs to the lymphoid tissues and tumors; (**II**) targeting of the dendritic cells, macrophages and other immune cells either through passive or active means; (**III**) uptake of the particles, escape from the endosome, antigen presentation and activation, activation of the innate immune system and activation of CD8^+^ and CD4^+^ T-cells using MHC Class I and II pathways; and (**IV**) activation and movement of the T-cells to the tumor for its recognition and elimination.

**Figure 3:**
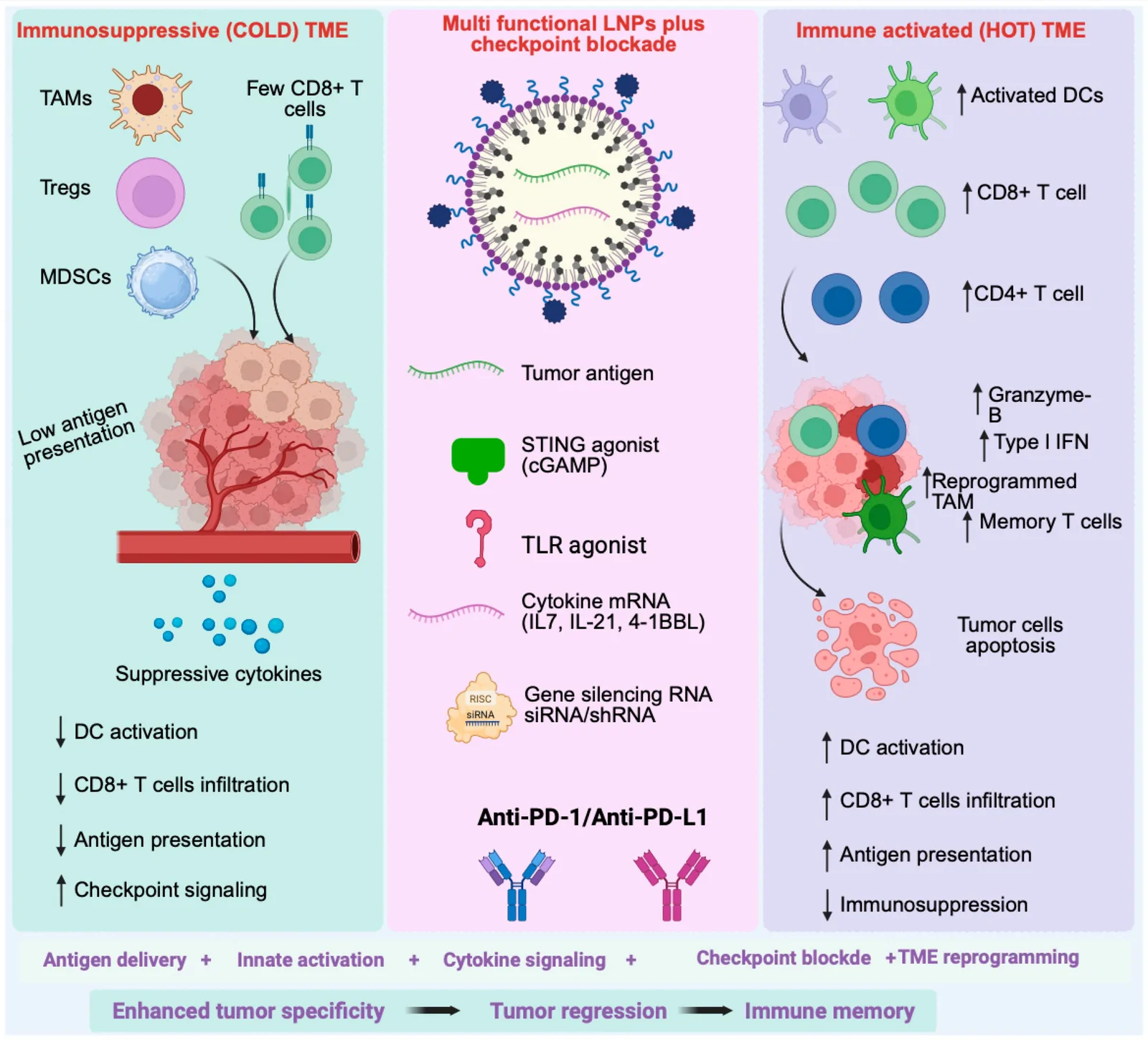
LNP combination immunotherapy and TME remodeling. “Cold” immunosuppressive TME includes immune cell subsets with suppressive properties, poor antigen presentation, and low T-cell infiltration and is associated with high checkpoint signaling activity. The use of multifunctional LNPs allows for delivery of tumor antigens, immune agonists, mRNAs encoding cytokines, and RNA for gene silencing. In combination with PD-1/PD-L1 checkpoint blockade therapy, such treatments provide a synergistic effect on anti-tumor activity. These mechanisms act together to stimulate dendritic cell activation, T-cell infiltration and functionality, repolarization of TAMs, tumor cell lysis, and T-cell memory development, thus transforming a “cold” TME into an activated “hot” one.

**Table 1:** Representative next-generation LNP engineering strategies relevant to cancer vaccination.

LNP Strategy	Engineering Feature	Key Outcomes	Reference
LQ089	AI-guided lipid design	Systemic delivery; activity comparable to SM-102	[6].
H9	AI-guided lipid design	~7.8-fold higher muscle expression vs. MC3	[7].
R6	Cell-optimized AI design	Macrophage-selective delivery	[7].
GL5-3	GCP-based, cholesterol-free LNP	Redirected mRNA expression from liver toward spleen	[9].
AMG1541	Rapidly biodegradable lipid	APC/dLN delivery; reduced hepatic exposure	[11].
Zn-9C-SCC-10	Biodegradable disulfide lipid	Lung-selective expression	[12].
ThrCo LNP	Cholesterol/PEG-free LNP	Splenic delivery; enhanced CD8^+^ responses	[13].
L@Mn-mRNA	Mn^2+^-mediated RNA condensation	Higher RNA loading; reduced lipid exposure	[15].
4A3-SC8	Controlled membrane disruption	Efficient expression with reduced membrane injury	[14].
Aromatic bioreducible LNPs	Disulfide-containing lipids	LN-selective delivery; memory responses	[16].

Abbreviations: AI, artificial intelligence; APC, antigen-presenting cell; GCP, guanidinocarbonyl-pyrrole; LNP, lipid nanoparticle; mRNA, messenger RNA; PEG, polyethylene glycol.

**Table 2:** Representative LNP-based cancer vaccine strategies and preclinical applications.

Study/Platform	Vaccine/Engineering Strategy	Key Immunological/Therapeutic Outcome	Reference
Large low-PEG LNPs	HPV16 E6/E7 mRNA; DC targeting	Strong CTL/memory responses; tumor regression at low dose	[19].
LiNx mRNA-LNP	Injectable nanofiber–hydrogel depot	Sustained CD8^+^ memory; durable single-dose anti-tumor activity	[22].
Mannose-targeted nanovaccine	Antigen mRNA + cGAMP; APC/STING targeting	Enhanced T-cell immunity and checkpoint-blockade response	[24].
Small circRNA-LNP	Stable antigen-encoding circRNA; LN targeting	Durable CD4^+^/CD8^+^ responses and tumor suppression	[9].
Peptide + cGAMP/MPLA NP	Neoantigen peptides + STING/TLR4 agonists	Enhanced cross-presentation, tumor control, and ICI response	[32]
AA15V LNP	Antigen-loaded MHC-I saRNA; tumor-cell targeting	Recruitment of antigen-specific CD8^+^ T-cells; tumor-growth inhibition	[36]

Abbreviations: APC, antigen-presenting cell; cGAMP, cyclic guanosine monophosphate–adenosine monophosphate; circRNA, circular RNA; CTL, cytotoxic T lymphocyte; DC, dendritic cell; HPV, human papillomavirus; ICI, immune-checkpoint inhibitor; LN, lymph node; LNP, lipid nanoparticle; MHC-I, major histocompatibility complex class I; MPLA, monophosphoryl lipid A; mRNA, messenger RNA; saRNA, self-amplifying RNA; STING, stimulator of interferon genes; TLR, Toll-like receptor.

**Table 3:** Immune-targeted and immunomodulatory LNP strategies for cancer immunotherapy.

Strategy/Platform	Target/Engineering Strategy	Key Immunological Effect	Reference
L17-F05	dLN APC targeting; tumor-antigen mRNA	Antigen presentation and STING-dependent innate activation	[50].
Mannose-modified LNP	Mannose-mediated DC targeting; antigen mRNA	Enhanced DC activation and antigen presentation	[39].
Antibody-capture LNP	Antibody-directed CD4^+^/CD8^+^ T-cell targeting	Selective systemic T-cell transfection	[51].
VISIT	Splenic DC targeting; antigen + IL-15/IL-15Rα mRNAs	Selective antigen-specific CTL activation and expansion	[9].
Triplet LNP	Tumor-localized IL-21, IL-7, and 4-1BBL expression	Increased cytotoxic T-cell infiltration and immune memory	[42].
Svg3 LNP	dLN/APC delivery; antigen + cGAS agonist	Enhanced CD8^+^ responses and reduced immunosuppression	[34].

Abbreviations: APC, antigen-presenting cell; cGAS, cyclic GMP–AMP synthase; CTL, cytotoxic T lymphocyte; DC, dendritic cell; dLN, draining lymph node; IL, interleukin; LNP, lipid nanoparticle; mRNA, messenger RNA; STING, stimulator of interferon genes; VISIT, vaccine-initiated selective T-cell modulation.

**Table 4:** Clinical translation of personalized cancer vaccines relevant to LNP-based immunotherapy.

Vaccine/Platform	Cancer	Treatment Strategy	Key Clinical Finding	Reference
Autogene cevumeran	Advanced solid tumors	≤20 neoantigens ± atezolizumab	Neoantigen-specific T-cell responses in 71% of evaluable patients	[55].
Intismeran autogene (mRNA-4157/V940)	High-risk melanoma	≤34 neoantigens + pembrolizumab	Reduced recurrence/death risk vs. pembrolizumab alone	[52].
Autogene cevumeran	Resected PDAC	Personalized neoantigens + atezolizumab/mFOLFIRINOX	Vaccine-expanded T-cells associated with delayed recurrence	[57].
Autogene cevumeran	PDAC	Personalized neoantigens; multimodal therapy	Durable neoantigen-specific CD8^+^ clones and prolonged recurrence-free survival in responders	[2].
Personalized neoantigen vaccine	High-risk ccRCC	Individualized multiepitope neoantigens	T-cell responses in all patients; clonotypes persisted ≤ 3 years	[25].
GNOS-PV02	Advanced HCC	Up to 40 personalized neoantigens	Neoantigen-specific CD4^+^/CD8^+^ responses and intratumoral T-cell trafficking	[58].

Abbreviations: ccRCC, clear-cell renal cell carcinoma; HCC, hepatocellular carcinoma; mFOLFIRINOX, modified folinic acid, fluorouracil, irinotecan, and oxaliplatin; PDAC, pancreatic ductal adenocarcinoma.

## Data Availability

No new data were created or analyzed in this study. Data sharing is not applicable to this article.

## References

[1] Sahin U., Schmidt M., Derhovanessian E., Cortini A., Vogler I., Omokoko T., Godehardt E., Attig S., Newrzela S., Grützner J. (2026). Individualized mRNA vaccines evoke durable T cell immunity in adjuvant TNBC. Nature.

[2] Sethna Z., Guasp P., Reiche C., Milighetti M., Ceglia N., Patterson E., Lihm J., Payne G., Lyudovyk O., Rojas L.A. (2025). RNA neoantigen vaccines prime long-lived CD8^+^ T cells in pancreatic cancer. Nature.

[3] Lee S., Park J., Jung H.N., Li S., Lin Z., Im H.-J. (2025). Magnetic Resonance Imaging-Based Quantification of Endosomal Escape Using Iron Oxide Nanoparticle-Loaded Lipid Nanoparticles. Adv. Healthc. Mater..

[4] Hussain M., Muglikar A., Brain D.E., Plant-Hately A.J., Liptrott N.J., McLoughlin D.M., Perrie Y. (2025). Redefining LNP composition: Phospholipid and sterol-driven modulation of mRNA expression and immune outcomes. RSC Pharm..

[5] Cheng M.H.Y., Zhang Y., Fox K., Leung J., Strong C., Kang E., Chen Y., Tong M., Bommadevara H., Jan E. (2025). Liposomal lipid nanoparticles for extrahepatic delivery of mRNA. Nat. Commun..

[6] Wang W., Chen K., Jiang T., Wu Y., Wu Z., Ying H., Yu H., Lu J., Lin J., Ouyang D. (2024). Artificial intelligence-driven rational design of ionizable lipids for mRNA delivery. Nat. Commun..

[7] Xu Y., Ma S., Cui H., Chen J., Xu S., Gong F., Golubovic A., Zhou M., Wang K.C., Varley A. (2024). AGILE platform: A deep learning powered approach to accelerate LNP development for mRNA delivery. Nat. Commun..

[8] Somu Naidu G., Rampado R., Sharma P., Ezra A., Kundoor G.R., Breier D., Peer D. (2025). Ionizable Lipids with Optimized Linkers Enable Lung-Specific, Lipid Nanoparticle-Mediated mRNA Delivery for Treatment of Metastatic Lung Tumors. ACS Nano.

[9] Zhang H., Liu D., Yang K., Liang Z., Li M. (2025). Ionizable guanidine-based lipid nanoparticle for targeted mRNA delivery and cancer immunotherapy. Sci. Adv..

[10] Holland R., Lam K., Jeng S., McClintock K., Palmer L., Schreiner P., Wood M., Zhao W., Heyes J. (2024). Silicon Ether Ionizable Lipids Enable Potent mRNA Lipid Nanoparticles with Rapid Tissue Clearance. ACS Nano.

[11] Rudra A., Gupta A., Reed K., Deik A., Min J., Mansour H.M.A., Nguyen Q.T.C., Danko A., Hu Y., Berger A. (2025). Degradable cyclic amino alcohol ionizable lipids as vectors for potent influenza mRNA vaccines. Nat. Nanotechnol..

[12] Chen Z., Yang Y., Qiu X., Zhou H., Wang R., Xiong H. (2024). Crown-like Biodegradable Lipids Enable Lung-Selective mRNA Delivery and Dual-Modal Tumor Imaging In Vivo. J. Am. Chem. Soc..

[13] Zhao Y., Tian Z., Wang J., Cui M., Cao Z., Liu P., Li R., Cai S., Hu Y., Ma Y. (2025). Replacing cholesterol and PEGylated lipids with zwitterionic ionizable lipids in LNPs for spleen-specific mRNA translation. Sci. Adv..

[14] Omo-Lamai S., Wang Y., Patel M.N., Milosavljevic A., Zuschlag D., Poddar S., Wu J., Wang L., Dong F., Espy C. (2025). Limiting endosomal damage sensing reduces inflammation triggered by lipid nanoparticle endosomal escape. Nat. Nanotechnol..

[15] Ma X., Liu S., Zhang S., Liu Z., Wang H., Luo W., Zu M., Qin H., Li Z., Zhong J. (2025). Engineering of mRNA vaccine platform with reduced lipids and enhanced efficacy. Nat. Commun..

[16] Yamagata H.M., Padilla M.S., Hamilton A.G., Swingle K.L., Thatte A.S., Ricciardi A.S., Agrawal A., Fitzgerald E., Poirier A.J., Geisler H.C. (2026). Liver-Detargeted Aromatic Bioreducible mRNA Lipid Nanoparticles Confer Lymph Node Tropism and Robust Antigen-Specific Immunity. J. Am. Chem. Soc..

[17] Jo S., Li L., Thakur C., Telfer K.A., Sultan H., Ohara R.A., He M., Nam G., Chen J., Ou F. (2026). mRNA vaccines engage unconventional pathways in CD8+ T cell priming. Nature.

[18] Kim Y., Park W., Kim S., Kim E.H., Choi J., Jang H., Ryu Y., Hwang J., Kim M., Cho Y.J. (2026). Dual-Targeting mRNA Cancer Vaccines for Simultaneous Antigen Presentation in Dendritic and Tumor Cells. ACS Nano.

[19] Xu X., An J., Bai D., Xu Y., Wu T., Yang H., Yan Y., Yang S., Wei Z., Yao Y. (2026). Large and low-PEG lipid nanoparticles enable efficient dendritic cell targeting for potent mRNA Cancer vaccines. J. Control. Release.

[20] Kim Y.A., Jeong H., Kim H., Lee S., Kim K.S., Na K. (2025). Lipid nanoparticles with prazole adjuvant to enhance the efficacy of mRNA cancer vaccines. J. Control. Release.

[21] Yang H., Gao Z., Zhou Y., Ge X., Pan X., Li J., Zhang Z., Jin X., Liu G., He Z. (2025). Tertiary amine N-oxide zwitterionic lipids facilitate muscle-selective mRNA vaccine delivery for enhancing cDC1-mediated antitumor efficacy. J. Control. Release.

[22] Zhu Y., Yao Z.-C., Li S., Ma J., Wei C., Yu D., Stelzel J.L., Ni B.Y.X., Miao Y., Van Batavia K. (2025). An mRNA lipid nanoparticle-incorporated nanofiber-hydrogel composite for cancer immunotherapy. Nat. Commun..

[23] Liu S., Wen Y., Shan X., Ma X., Yang C., Cheng X., Zhao Y., Li J., Mi S., Huo H. (2024). Charge-assisted stabilization of lipid nanoparticles enables inhaled mRNA delivery for mucosal vaccination. Nat. Commun..

[24] Jin C., Zhang Y., Li B., Gao T., Wang B., Hua P. (2024). Robust anti-tumor immunity through the integration of targeted lipid nanoparticle-based mRNA nanovaccines with PD-1/PD-L1 blockade. Mater. Today Bio.

[25] Braun D.A., Moranzoni G., Chea V., McGregor B.A., Blass E., Tu C.R., Vanasse A.P., Forman C., Forman J., Afeyan A.B. (2025). A neoantigen vaccine generates antitumour immunity in renal cell carcinoma. Nature.

[26] Garfinkle E.A.R., Perales-Linares R., Gimple R.C., Livingstone A.J., Roberts K.F., Butt O.H., Goedegebuure S.P., McLellan M.D., Chang G.S., Hundal J. (2026). Adjuvant personalized multivalent neoantigen DNA vaccination for MGMT unmethylated glioblastoma: A phase 1 trial. Nat. Cancer.

[27] Lin G., Tang Y.-L., Fu Z., Chen R., Liu Y., Liu Z., Kuang X., Sun J., Zhao J., Zhang Y. (2025). Enhancing protective immunity against SARS-CoV-2 with a self-amplifying RNA lipid nanoparticle vaccine. J. Control. Release.

[28] Zhang Y., Liu X., Shen T., Wang Q., Zhou S., Yang S., Liao S., Su T., Mei L., Zhang B. (2025). Small circular RNAs as vaccines for cancer immunotherapy. Nat. Biomed. Eng..

[29] Alshehry Y., Liu X., Zhang Y., Zhu G. (2025). Investigation of the impact of lipid nanoparticle compositions on the delivery and T cell response of circRNA vaccine. J. Control. Release.

[30] Cui X., Vervaeke P., Gao Y., Opsomer L., Sun Q., Snoeck J., Devriendt B., Zhong Z., Sanders N.N. (2024). Immunogenicity and biodistribution of lipid nanoparticle formulated self-amplifying mRNA vaccines against H5 avian influenza. npj Vaccines.

[31] Ye T., Zhong Z., Cappellesso F., Deswarte K., Chen Y., Lauwers H., De Lombaerde E., Gontsarik M., Lienenklaus S., Van Lysebetten D. (2024). CO-DELIVERY of glutamic acid-extended peptide antigen and imidazoquinoline TLR7/8 agonist via ionizable lipid nanoparticles induces protective anti-tumor immunity. Biomaterials.

[32] Baljon J.J., Kwiatkowski A.J., Pagendarm H.M., Stone P.T., Kumar A., Bharti V., Schulman J.A., Becker K.W., Roth E.W., Christov P.P. (2024). A Cancer Nanovaccine for Co-Delivery of Peptide Neoantigens and Optimized Combinations of STING and TLR4 Agonists. ACS Nano.

[33] Luo Y., Zhou S., Song Y., Huang W.-C., Wilding G.E., Jablonski J., Quinn B., Lovell J.F. (2025). Iterative selection of lipid nanoparticle vaccine adjuvants for rapid elicitation of tumoricidal CD8^+^ T cells. Bioact. Mater..

[34] Zhou S., Liang Y., Hao Y., Wang Q., Xu Y., Su T., Cheng F., Zhu G. (2025). Combined cancer immunotherapy with lipid nanoparticle delivery of oligo-based cGAS-agonistic adjuvant and peptide or mRNA vaccines. Mol. Ther. Nucleic Acids.

[35] Chillara M., Zhao W., Dordick J.S., Przybycien T. (2026). Effect of transcript length, temperature, and lyophilization on mRNA-lipid nanoparticle stability. J. Pharm. Sci..

[36] Xue Y., Hou X., Zhong Y., Zhang Y., Du S., Kang D.D., Wang L., Wang C., Li H., Wang S. (2025). LNP-RNA-mediated antigen presentation leverages SARS-CoV-2-specific immunity for cancer treatment. Nat. Commun..

[37] Zhou Y., Xie Y., Qi J., Hu J., Yang Y., Deng F., Zhu Y., Deng W., Mei L., Goldys E.M. (2026). A leucine derivate-adjuvanted LNP vaccine enhances antitumor immunity through mTOR activation and metabolic reprograming in dendritic cells. Biomaterials.

[38] Kim C., Lee Y., Lee H., Kim B.-S. (2025). Advances in mRNA-Lipid Nanoparticle Engineering for Immune Cell Targeting and Immune Modulation. Small Methods.

[39] Wang S., Zheng J., Zhou J., Jiang W., Chen Z., Wu X., Guo B., Wu Y., Yang F. (2025). Engineered lipid nanoparticles with synergistic dendritic cell targeting and enhanced endosomal escape for boosted mRNA cancer vaccines. Mater. Today Bio.

[40] Muro R., Wang S., Ito-Kureha T., Huynh H.H., Kobiyama K., Sugita T., Okamoto K., Nakano K., Okamura T., Kubo M. (2026). A distinct antigen presentation pathway drives potent T cell immunity in lipid nanoparticle-based mRNA vaccines. Sci. Adv..

[41] Zhang C., Stewart W., Teng Y., Hu B., Xu X., Zhang X.Q. (2025). mRNA-Based Vaccination Drives in Vivo Dendritic Cell Reprogramming and Selective Cytotoxic T Lymphocyte Modulation for Enhanced Antitumor Immunity. ACS Nano.

[42] Hamouda A.E.I., Filtjens J., Brabants E., Kancheva D., Debraekeleer A., Brughmans J., Jacobs L., Bardet P.M.R., Knetemann E., Lefesvre P. (2024). Intratumoral delivery of lipid nanoparticle-formulated mRNA encoding IL-21, IL-7, and 4-1BBL induces systemic anti-tumor immunity. Nat. Commun..

[43] Abdellatif A., Monteiro C., Bernades M., Jofre L. (2024). Microconfined High-Pressure Transcritical Channel Flow Database: Laminar, Transitional & Turbulent Regimes. Sci. Data.

[44] Luo Y., Li Q., Zhou S., Oh H., Jablonski J., Song Y., Su Y., Wu Y., Zhu H., Ortega J. (2026). Lipid nanoparticles that co-deliver poly(I:C) and short peptide antigens elicit anti-tumor responses with vaccination. Biomaterials.

[45] Zhao N., Liu Z., Zhao Q., Feng Z., Wang X., Yang X., Zhang M., Zhong Y., Xiao Y., Chen X. (2026). Lipid nanoparticle-mediated co-delivery of IL-33 mRNA and STING agonist modulates the breast cancer microenvironment and potentiates immunotherapy. J. Nanobiotechnology.

[46] Fournier C., Mercey-Ressejac M., Derangère V., Al Kadi A., Rageot D., Charrat C., Leroy A., Vollaire J., Josserand V., Escudé M. (2025). Nanostructured lipid carriers based mRNA vaccine leads to a T cell-inflamed tumour microenvironment favourable for improving PD-1/PD-L1 blocking therapy and long-term immunity in a cold tumour model. eBioMedicine.

[47] Liang Q., Peng Y., Zhao L., Yang Z., Chen X., Li J., Wu Z., Chen K., Zhou B., Liu Y. (2026). Pulmonary macrophage-targeted RNAi and mRNA co-delivery via SORT lipid nanoparticles enhances immunotherapy in lung cancer. J. Control. Release.

[48] Gao Y., Li J., Xu M., Liu Y., Guo L., Li J. (2026). Reprogramming tumour-associated macrophages in breast cancer via si-FOXM1-loaded lipid nanoparticles enhances immune checkpoint inhibitor efficacy. Br. J. Pharmacol..

[49] Xu Z., Qiu H.-W., Xu Y., Wang J., Cao F., Wang Y., Guan Y., Xiao Z., Zha G.-F., Pang J. (2026). Fluorinated lipid nanoparticles enable in vivo CAR-macrophage therapy in solid tumor and enhance anti PD-L1 immunotherapy. Biomaterials.

[50] Kubara K., Yamazaki K., Miyazaki T., Kondo K., Kurotaki D., Tamura T., Suzuki Y. (2024). Lymph node macrophages drive innate immune responses to enhance the anti-tumor efficacy of mRNA vaccines. Mol. Ther..

[51] Chen M.Z., Yuen D., McLeod V.M., Yong K.W., Smyth C.H., Herling B.R., Payne T.J., Fabb S.A., Belousoff M.J., Algarni A. (2025). A versatile antibody capture system drives specific in vivo delivery of mRNA-loaded lipid nanoparticles. Nat. Nanotechnol..

[52] Weber J.S., Carlino M.S., Khattak A., Meniawy T., Ansstas G., Taylor M.H., Kim K.B., McKean M., Long G.V., Sullivan R.J. (2024). Individualised neoantigen therapy mRNA-4157 (V940) plus pembrolizumab versus pembrolizumab monotherapy in resected melanoma (KEYNOTE-942): A randomised, phase 2b study. Lancet.

[53] Feng K., Zhang X., Li J., Han M., Wang J., Chen F., Yi Z., Di L., Wang R. (2025). Neoantigens combined with in situ cancer vaccination induce personalized immunity and reshape the tumor microenvironment. Nat. Commun..

[54] Li Y., Fang M., Yu H., Wang X., Xue S., Jiang Z., Huang Z., Rong S., Wei X., Lu Z. (2025). Neoantigen enriched biomimetic nanovaccine for personalized cancer immunotherapy. Nat. Commun..

[55] Lopez J., Powles T., Braiteh F., Siu L.L., LoRusso P., Friedman C.F., Balmanoukian A.S., Gordon M., Yachnin J., Rottey S. (2025). Autogene cevumeran with or without atezolizumab in advanced solid tumors: A phase 1 trial. Nat. Med..

[56] Carlino M.S., Khattak A., Meniawy T., Ansstas G., Taylor M.H., Kim K.B., McKean M., Long G.V., Sullivan R.J., Faries M. (2026). Three-Year Update of a Randomized Phase IIb Study of the Individualized Neoantigen Therapy Intismeran Autogene (mRNA-4157, V940) Plus Pembrolizumab Versus Pembrolizumab in Resected Melanoma. JCO Oncol. Adv..

[57] Rojas L.A., Sethna Z., Soares K.C., Olcese C., Pang N., Patterson E., Lihm J., Ceglia N., Guasp P., Chu A. (2023). Personalized RNA neoantigen vaccines stimulate T cells in pancreatic cancer. Nature.

[58] Yarchoan M., Gane E.J., Marron T.U., Perales-Linares R., Yan J., Cooch N., Shu D.H., Fertig E.J., Kagohara L.T., Bartha G. (2024). Personalized neoantigen vaccine and pembrolizumab in advanced hepatocellular carcinoma: A phase 1/2 trial. Nat. Med..

[59] Hwang Y.H., Shepherd S.J., Kim D., Mukalel A.J., Mitchell M.J., Issadore D.A., Lee D. (2025). Robust, Scalable Microfluidic Manufacturing of RNA-Lipid Nanoparticles Using Immobilized Antifouling Lubricant Coating. ACS Nano.

[60] Devos C., Udepurkar A., Sagmeister P., Hodlewsky A.S., Chen J., Hatas A., Ostrovsky N., Al-Jazrawe M., Ren J.I., Liu A.Y. (2025). Manufacturing mRNA-Loaded Lipid Nanoparticles with Precise Size and Morphology Control. ACS Nano.

[61] Wu W., Oliveira L.T., Jain A., Karpov Y., Olsen K., Wu Y., Gopalakrishna Panicker R.K. (2025). Process development of tangential flow filtration and sterile filtration for manufacturing of mRNA-lipid nanoparticles: A study on membrane performance and filtration modeling. Int. J. Pharm..

[62] Ojalvo L.S., Posadas T., Chattopadhyay N., Porichis F., Shah S., Cristescu R., Rao A. (2026). Intismeran Autogene Therapy: An End-to-End Pathway From Tumor Tissue to Individualized Neoantigen Treatment for Patients with Cancer. JCO Oncol. Adv..

[63] Cho S., Lee J., Lee Y.-M., Nam J.-H. (2026). Take Five: Harmonization in personalized cancer vaccines for cancer immunotherapy. Exp. Mol. Med..

[64] Chan A., Kirtane A.R., Qu Q.R., Huang X., Woo J., Subramanian D.A., Dey R., Semalty R., Bernstock J.D., Ahmed T. (2025). Designing lipid nanoparticles using a transformer-based neural network. Nat. Nanotechnol..

[65] Su L.-J., Wang N.-N., Luo R., Ji Z.-H., Gu H., Yang C., Xu M.-X., Zhang J., Chen Q., Yu M.-Z. (2026). Artificial intelligence-guided design of LNPs for in vivo targeted mRNA delivery via analysis of the spatial conformation of ionizable lipids. Nat. Biomed. Eng..

